# Excellent interobserver agreement and steep learning curve for target volume delineation for stereotactic arrhythmia radioablation using a commercial software

**DOI:** 10.1093/europace/euaf122

**Published:** 2025-06-16

**Authors:** Robert Rademaker, Simon Cirkel, Sharif Omara, Frank J W M Dankers, Marek Sramko, J Solana Munoz, Yesim S Kaya, Rachel M A Ter Bekke, Luis Schiappacasse, Cheryl Teres, Karolien Verhoeven, Etienne Pruvot, Coen R N Rasch, Katja Zeppenfeld

**Affiliations:** Department of Cardiology, Leiden University Medical Center, P.O. Box 9600, Leiden 2300 RC, The Netherlands; Willem Einthoven Center of Arrhythmia Research and Management, Leiden, The Netherlands, Aarhus, Denmark; Department of Cardiology, Leiden University Medical Center, P.O. Box 9600, Leiden 2300 RC, The Netherlands; Willem Einthoven Center of Arrhythmia Research and Management, Leiden, The Netherlands, Aarhus, Denmark; Department of Cardiology, Leiden University Medical Center, P.O. Box 9600, Leiden 2300 RC, The Netherlands; Willem Einthoven Center of Arrhythmia Research and Management, Leiden, The Netherlands, Aarhus, Denmark; Department of Radiation Oncology, Leiden University Medical Center, Leiden, The Netherlands; Department of Cardiology, Institute for Clinical and Experimental Medicine, Prague, Czech Republic; Department of Cardiology, Lausanne University Hospital, Lausanne, Switzerland; Department of Cardiology, Maastricht University Medical Center+ (MUMC+), Cardiovascular Research Institute Maastricht (CARIM), Maastricht University, Maastricht, The Netherlands; Department of Cardiology, Maastricht University Medical Center+ (MUMC+), Cardiovascular Research Institute Maastricht (CARIM), Maastricht University, Maastricht, The Netherlands; Department of Radiation Oncology, Lausanne University Hospital, Lausanne, Switzerland; Department of Cardiology, Lausanne University Hospital, Lausanne, Switzerland; Department of Radiation Oncology (Maastro), GROW-Research Institute for Oncology and Reproduction, Maastricht University Medical Center+, Maastricht, The Netherlands; Department of Cardiology, Lausanne University Hospital, Lausanne, Switzerland; Department of Radiation Oncology, Leiden University Medical Center, Leiden, The Netherlands; Department of Cardiology, Leiden University Medical Center, P.O. Box 9600, Leiden 2300 RC, The Netherlands; Willem Einthoven Center of Arrhythmia Research and Management, Leiden, The Netherlands, Aarhus, Denmark

**Keywords:** Ventricular tachycardia, Ablation, Stereotactic arrhythmia radioablation, STAR, Interobserver variability in imaging and EAM merging

## Abstract

**Aims:**

Stereotactic arrhythmia radioablation (STAR) has emerged as bail-out treatment for ventricular tachycardia (VT). Accurate, reproducible, and easy-to-use data transfer from electroanatomical mapping (EAM) systems to radiotherapy planning CT is desirable. We aim to evaluate interobserver variability, ease of use, and learning curve for EAM based target volume (CardTV-EP_inv_) creation and transfer using available software packages.

**Methods and results:**

In patients considered for STAR, CardTV-EP_inv_ were created using ADAS and Slicer3D for workflow comparison. Four CardTV-EP_inv_ (clinically targeted volume and three mock targets) were created by an experienced operator and a 2nd-year medical student, based on endocardial EAM tags indicating VT substrate location. CardTV-EP_inv_ sizes, Hausdorff distances (HDs), and workflow duration were measured to assess interobserver variability and learning curve. Agreement between CardTV-EP_inv_ was high using ADAS and Slicer3D workflows (HD 3.64 mm [2.7–4.5]). ADAS workflow was faster and more robust (ADAS 26 min [24–29] vs. Slicer3D 65 min [61–70], *P* < 0.001; system crashes: ADAS 0 vs. Slicer3D 7). In 20 patients (80% non-ischaemic cardiomyopathy, LVEF 35 ± 14%), 80 CardTV-EP_inv_ were created using ADAS. CardTV-EP_inv_ size was similar for both observers (11.8 mL [10.1–13.7] vs. 10.7 mL [9.6–11.8], *P* = 0.17), with high interobserver agreement (HD 1.68 mm [1.45–1.96]; 95th percentile HD < 4.8 mm [3.5–5.7]). Linear regression showed a steep learning curve for the student (*P* = 0.01).

**Conclusion:**

CardTV-EP_inv_ creation showed excellent interobserver agreement and was faster and more robust using ADAS than 3D slicer. The steep learning curve appears clinically relevant given the limited use of STAR even in high-volume VT ablation centres.

## Graphical Abstract

**Graphical Abstract:**
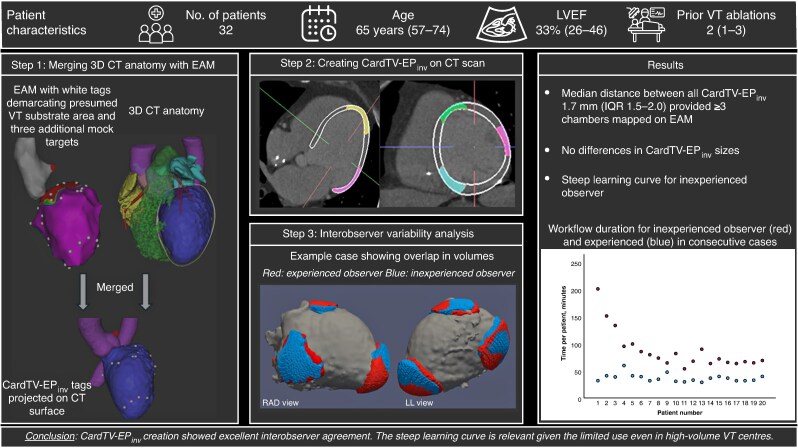

What’s new?

- This is the first study to evaluate the interobserver variability using open-source and commercially available software packages for the transfer of CardTV-EP_inv_ to the planning software for stereotactic arrhythmia radioablation (STAR).
- Cardiac target volume creation is faster and more robust using the commercially available ADAS compared to the open-source Slicer 3D software, with an excellent interobserver agreement for both single- and multicentre electroanatomical mapping data, provided that recommended mapping standards are applied.
- The steep learning curve for inexperienced operators using ADAS is clinically relevant considering the limited use of STAR, even in high-volume VT ablation centres.

## Introduction

Catheter ablation is one of the cornerstones in the treatment of scar-related ventricular tachycardia (VT).^1–3^ Outcomes are favourable for subendocardial or subepicardial substrate locations.^1,4–9^ One important limitation of the technique is the inability to reach deep intramural substrates, or those protected by (epicardial) fat or calcifications.^10–13^ Since its first report, stereotactic arrhythmia radioablation (STAR) has emerged as a bail-out treatment option for patients with inaccessible substrates.^14,15^

Stereotactic arrhythmia radioablation requires a close collaboration between electrophysiologists and radiation-oncologists. The delineation of the inaccessible VT substrate [=cardiac target volume (CardTV)] is mainly based on invasive endocardial or endo- and epicardial electroanatomical mapping (EAM) data provided by the electrophysiologist, which needs to be extrapolated to a transmural volume, referred to as CardTV-EP_inv_.^16^ 3D EAM data can be co-registered with a computed tomography (CT) scan and the delineated CardTV-EP_inv_ can be transferred to the CT, which is used for radiation treatment planning. Different workflows for CardTV-EP_inv_ delineation, co-registration, and target data transfer have been proposed.^17–21^ Workflows have utilized either the open-source Slicer 3D software (3D to 3D registration),^17,18,22^ or in-house-developed software packages (2D to 3D registration)^22,23^ or the 17-segment American Heart Association (AHA) model,^20^ while others have manually transferred EAM data to the 2D CT slices based on eyeballing.^19,24^ The quality of the available 3D EAM data has been shown to impact the interobserver agreement for the transferred CardTV-EP_inv_.^18,21^

Since the use of STAR remains limited, even in high-volume VT ablation centre with >50 complex VT ablations per year,^2^ there is a need for an accurate, reproducible, and robust data transfer workflow, which is easy to learn and/or already used by electrophysiologists. The ADAS 3D anatomy segmentation tool is a commercially available software package (ADAS 3D Medical SL), which is used as a (pre-)procedural image integration tool, in both atrial and ventricular ablations.^25^ The use of ADAS in CardTV-EP_inv_ delineation has been only case-reported.^26^ Slicer 3D workflow uses open-source software and has been reported as a method of 3D-to-3D transfer of mapping data to CT with a high interobserver agreement.^17,21,22,27^

The aims of this study are threefold: to evaluate^1^ the intraobserver variability, ease of use, and workflow duration for available software packages;^2^ the interobserver agreement for CardTV-EP_inv_ creation and transfer using (i) single centre data and (ii) multicentre data of STAR-treated patients from the STOPSTORM.eu consortium^28^; and^3^ the learning curve for CardTV-EP_inv_ creation and transfer.

## Methods

### Patient population

The study population consisted of patients who underwent EAM and catheter ablation for VT using the CARTO mapping system (Biosense Webster Inc., CA, USA) and were considered potential candidates for STAR bail-out therapy based on the substrate properties known to be difficult to control with ablation. All patients were identified as potential candidates for STAR during the ablation procedure, based on the acute procedural outcome (partial procedural success, failure to completely eliminate the VT substrate). In each patient, the potential target area was demarcated on the EAM after the procedure. Fortunately, not all patients needed STAR as bail-out therapy after ablation. Since the planning CT is made immediately prior to radiation, only the patients who underwent STAR had a planning CT available. Patients were treated at the Leiden University Medical Center (LUMC), Leiden, The Netherlands, the Lausanne University Hospital (CHUV), Lausanne, Switzerland, the Maastricht University Medical Center (MUMC+)/Maastro, Maastricht, The Netherlands, or the Institute for Clinical and Experimental Medicine (IKEM), Prague/University Hospital of Ostrava, Ostrava, Czech Republic between January 2018 and March 2024. Supplementary material online, *Figure S1* visualizes the patient cohorts.

### CT acquisition

All ECG-gated CT scan with contrast was acquired in diastole and inspiration breath-hold. The slice thickness of the ECG-gated CT scans was 0.5 mm. Radiotherapy 3D contrast CTs and 4D respiratory-gated CTs were acquired in free breathing (in the same frame of reference). Target delineations were expanded to account for respiratory motion using the 4D CT phases. Radiotherapy treatment planning was performed on 3D CT (*n* = 7), or 4D CT average reconstruction (*n* = 4). The slice thickness and spacing between slices were 2 mm for all cases, and the in-plane pixel spacing was 1 ± 0.1 mm in left–right and anterior–posterior direction for all cases.

### Workflow for cardiac target volume creation

Target areas were indicated on the (endocardial) surface of the EAM in Carto using pre-defined tags that encircle the area of the inaccessible substrate, defined as area of interest (AOI). All EAM data were exported from the Carto system for offline use.

The workflow for creating the CardTV-EP_inv_ using Slicer 3D has been described previously.^21^ Briefly, the endocardial EAM surface data in polygon .mesh data and the tags created on the EAM were imported in Slicer 3D, where the cardiac anatomy was segmented from the CT scan. Using custom Python plugins, the EAM points were projected on the 3D anatomy. Then, using the projected points on the LV surface, the CardTV-EP_inv_ was determined by creating perpendicular lines from the endocardial surface to the epicardial surface. These lines were then connected to form one volume (CardTV-EP_inv_) based on the 2D target area demarcated by the projected EAM points.

Using ADAS, the cardiac anatomy [aorta, left ventricle (LV), left main coronary artery, right ventricle (RV), and pulmonary artery (PA)] was extracted from the cardiac CT and projected as a 3D model using the Heart Anatomy Extraction tool. This was done with either the auto-segmentation tool or, when auto-segmentation did not yield satisfactory results, for example, due to artefacts from cardiac implantable devices, threshold segmentation was used. Anatomical structure contours were verified to be correct with the 2D slices in all three axes. The left ventricular wall thickness was determined by manually contouring the endo- and epicardial surface. The space between the contours (the LV wall) was converted into a 3D structure. After importing the EAM data into ADAS, the 3D mapping data were merged with the 3D CT model using all available EAM structures and distinct landmarks (LV/RV/aorta/left main coronary/pulmonary artery, etc.) by manually translating and rotating the structures and aligning the landmark points where available. The target area tags were projected on the 3D LV endocardial surface. In ADAS, EAM points were automatically projected to the closest endocardial contour. In the Slicer 3D workflow, this option is not available. Using the segment creation tool in ADAS 3D, the AOI was converted by the operator into a transmural 3D volume (CardTV-EP_inv_) in the 3D LV wall. This was done by first connecting the tags on the endocardial surface and then creating perpendicular lines from the endocardial circle to the closest epicardial border, or the RV endocardial contours for septal sites. See Supplementary material for a detailed workflow description. The CardTV-EP_inv_ was created on both the cardiac (diastolic ECG-gated) CT scan and the radiation-oncology (non-ECG-gated) planning CT scan (where available) to determine agreement between volumes using these different CT acquisition sequences.

In addition to the clinical AOI, three remote areas of a potentially inaccessible VT substrate location were indicated by tags on a separate map to create mock CardTV-EP_inv_ to determine the influence of substrate location on interobserver variability. These areas were located in basal anterior segments (LV summit region, AHA segment 1), mid-septal (AHA segments 8 and 9), mid-lateral (AHA segments 11 and 12), and apico-inferior (AHA segment 15) segments representing common substrate locations in patients undergoing STAR.^19,21,24,29^

The time needed for each step and the performance of the software packages (e.g. software crashes) were noted.

### Intraobserver variability, ease of use, and workflow time for available software packages

For the evaluation of the intraobserver variability and ease of use, both workflows were followed by operator #1 (R.R.) highly experienced with both software packages. The patients used for this analysis consisted of the LUMC patients treated by STAR, with only the clinical target volume analysed, without mock volumes. The same target area indicated by the pre-defined tags on the 3D EAM was used to create CardTV-EP_inv_. The number of software crashes during the creation of the CardTV-EP_inv_ was registered. A software crash was defined as a non-intended shutdown of the software program with consequential data loss. The time lost due to a software crash was not included in the total workflow time.

The created CardTV-EP_inv_ were exported from ADAS and Slicer 3D in .vtk format and imported in Slicer 3D for comparison. Using a built-in tool in Slicer 3D (Segment Comparison), the volumes of the CardTV-EP_inv_ and the mean- and 95% Hausdorff distance (HD) between each pair of CardTV-EP_inv_ were determined. The HD is the greatest of all distances from a point of one surface to a point on the co-registered surface. The HD was calculated for each outer surface point on the created volumes.^21^ The mean HD is defined as the average of all distances, and the 95% HD is the 95th percentile of the ordered distance (95% of all points are within this distance).^21^

### Interobserver agreement for CardTV-EP_inv_ creation and transfer using (i) single centre data and (ii) multicentre data of stereotactic arrhythmia radioablation-treated patients from the STOPSTORM.eu consortium

To evaluate the interobserver variability of CardTV-EP_inv_ creation and transfer using high quality data as previously defined,^21^ datasets from a single high-volume centre (LUMC) were used. This patient cohort consisted of all patients treated for VT by ablation in the LUMC (*n* = 20) and who were considered potential candidates for STAR. Fortunately, not all patients required STAR after ablation. For each case, mapping data of at least three structures and/or landmarks and ECG-gated CT scans with contrast were available. In addition to the clinical target area, three additional mock areas were created to determine the influence of the substrate location on the interobserver agreement.

To determine the potential impact of multicentre data (e.g. variation in mapping density, variation in number and type of structures/landmarks mapped) on the interobserver variability, datasets from four centres involved in the European prospective STOPSTORM.eu consortium were used.^28^ All included patients were considered candidates for STAR treatment and had the clinical target area indicated on the 3D EAM. The single centre patients who underwent STAR after ablation (*n* = 7) were also included in the multicentre data analysis. The clinically treated CardTV-EP_inv_ created by observer #1 in the intraobserver agreement analysis (ADAS/Slicer comparison) were re-used in the multicentre (ADAS/ADAS) comparison.

Two observers [observer #1, highly experienced in creating CardTV-EP and observer #2 (S.C.) inexperienced] performed the entire workflow separately using ADAS. The volumes of the CardTV-EP and the mean- and 95% HD between each pair of CardTV-EP were determined.

To determine the potential variability in volumes using an ECG-gated CT or a non-gated radiotherapy planning CT, the clinical CardTV-EP_inv_ was created using ADAS on both CT scans.

The analysis included four steps of potential interobserver variability in the transfer of EAM data to the CT scan:

1. Segmentation of the 3D anatomy from the 2D CT slices;
2. Co-registration of the EAM data with the 3D CT anatomy;
3. Location of the EAM target area tags on the CT endocardial surface when automatic projection of the EAM points to the endocardial surface is not available (i.e. in the Slicer3D workflow); and
4. Creation of the final CardTV-EP_inv_ including the direction of transmurality (e.g. at the RV insertion) and the involvement of adjacent structures (e.g. papillary muscles).

### Learning curve for an inexperienced observer

To determine the learning curve of an inexperienced observer, the time needed for the segmentation of the anatomy, the co-registration of EAM data with the CT anatomy and for the creation of the CardTV-EP_inv_ was measured. The inexperienced observer, a medical student without any prior experience with 3D mapping data, cardiac CT (CCT) reading, and the use of the software package, received a written description of the workflow and a one-time demonstration.

### Statistical analysis

Continuous variables are reported as median with interquartile range (IQR) or mean ± standard deviation, when appropriate. Data were compared using the Student’s *t*-test, Mann–Whitney *U* test, and one-way analysis of variance (ANOVA), when appropriate. Linear regression models were calculated using ANOVA. Categorical variables were compared with the χ^2^ test and Fisher’s exact test, when appropriate. *P*-values below 0.05 were considered statistically significant. All statistical analyses were performed in SPSS version 27.0 (IBM Corporation, Armonk, New York).

### Ethical approvement

All used patient data were (pseudo)anonymized: imaging series and EAM were named after the centre, and a follow-number was given per patient (e.g. LUMC_1). Patient baseline characteristics data were exported by the treating physician and coded in a similar manner. Ethical approval for data usage was obtained through the local ethical committee (non-WMO, METc Leiden University, Leiden, The Netherlands, reference W22_193 # 22.241) for the single centre patients and through the STOPSTORM.eu ethical approval for the multicentre patients (non-WMO, Amsterdam UMC, Amsterdam, The Netherlands, reference W22_193 # 22.241).

## Results

### Patient population

A total of 32 patients from four centres were included (median age 65 years [IQR 57–74], 91% male, 70% non-ischaemic cardiomyopathy (NICM), median LVEF 33% [IQR 26–46], median 2 [IQR 1–3] prior VT ablations). In 20/32 patients, an ECG-gated CT scan was available and in 19/32 a non-gated planning CT was also available. Among all scans, left ventricular contrast was present in 31/32 patients. In 16/32 patients, both LV and RV contrast was present. All patients had an LV EAM available, and in 24/32 patients at least three structures/landmarks were mapped (including LV, RV, aorta, pulmonary artery, left atrium, ostium of the left main coronary artery). For the 19 patients with an available radiotherapy planning CT and CardTV-EP_inv_ demarcated by endocardial tags, the target areas were lateral,^6^ septal,^6^ anterior/apical,^3^ basal anterior,^2^ and inferior.^2^ Nineteen of the 32 patients were included in the STOPSTORM.eu registry. See *Table 1* for details.

**Table 1:** Patient characteristics, imaging protocols, and electroanatomical mapping structures

	All patients	Single centre patients	Multicentre patients
	*n* = 32	*n* = 20	*n* = 19^a^
Patient characteristics			
Age, years	65 [57–74]	65 [57–75]	65 [56–73]
Male	29 (91)	19 (95)	17 (90)
NICM	22 (70)	16 (80)	9 (47)
LVEF, %	33 [26–46]	32 [26–44]	32 [24–46]
Prior VT ablations	2 [1–3]	1 [1–3]	3 [2–4]
Imaging			
LV contrast	15 (47)	9 (45)	8 (42)
LV + RV contrast	16 (50)	11 (55)	10 (53)
No contrast	1 (3)	0 (0)	1 (5)
ECG-gated CT	20 (63)	20 (100)	7 (37)
Non-gated planning CT	19 (59)	7 (35)	19 (100)
EAM structures			
Left ventricle	32 (100)	20 (100)	19 (100)
Aorta	24 (75)	20 (100)	11 (58)
Right ventricle	16 (50)	11 (55)	10 (53)
Left main coronary artery	19 (59)	19 (95)	7 (37)
Pulmonary artery	10 (31)	8 (40)	6 (32)
Left atrium	4 (13)	0 (0)	4 (21)
Three or more structures mapped	24 (75)	20 (100)	11 (53)

Numbers are provided as *n* (%) and median [IQR].

CT, computed tomography; EAM, electroanatomical map; ECG, electrocardiogram; LV, left ventricle; LVEF, left-ventricular ejection fraction; NICM, non-ischaemic cardiomyopathy; RV, right ventricle; VT, ventricular tachycardia.

^a^Including 7 LUMC patients (single centre).

### Intraobserver variability, ease of use, and workflow time for available software packages

Datasets of seven patients [67 ± 12 years, four NICM, median LVEF 26% (range 19–42), median 3 prior ablation (range 1–6)] who underwent STAR at the LUMC were processed by the experienced operator using ADAS and Slicer 3D. All patients had available EAM data of the endocardial LV, the aorta, and the position of the left main coronary artery ostium (LM). RV endocardial mapping was performed in 4/7. All patients had ECG-gated cardiac CT with contrast. In all patients, the clinical AOI was indicated by tags and was located at the (basal) septum (*n* = 2), the basal anterior (*n* = 1), lateral (*n* = 2), and apical (*n* = 2) LV.

The CardTV-EP_inv_ created in Slicer 3D were larger compared to those created in ADAS (median 50.2 mL [IQR 33.4–84.8] vs. 36.0 mL [IQR 14.4–50.3], median volume difference 21.8 mL [IQR 3.4–28.9], *P* < 0.05). The average HD between volumes was 3.6 mm [2.7–4.5], and the 95th percentile distance was 9.0 mm (6.72 [IQR 6.7–13.0]). The differences in volume size in the Slicer 3D workflow were explained by an expansion of the volume into the blood pool for septal substrates and into the blood pool and extracardiac structures for the basal, lateral, and inferior target areas. In all cases, the entire volume created in ADAS was overlapped by the volume created in Slicer3D. See examples in Supplementary material online, *Figure S3*. Duration of the Slicer 3D workflow was significantly longer than that with ADAS (65 [IQR 61–70] vs. 26 [24–29] min, respectively, *P* < 0.001). Seven total software crashes occurred in four cases while using the Slicer 3D workflow but none with the ADAS workflow. *Table 2* provides the details of the workflow comparison. Considering the longer workflow duration and the high number of software crashes using Slicer 3D, the interobserver variability analysis for single and multicentre data was only performed with ADAS.

**Table 2:** Comparison between ADAS and Slicer 3D workflow (experienced operator)

	ADAS	Slicer 3D	*P*-value
	*n* = 7	*n* = 7	
CardTV-EP volumes			
Volume, mL	36.0 [14.4–50.3]	50.2 [33.4–84.8]	0.01
Difference in volumes, mL [IQR]	21.8 [IQR 3.4–28.9]		0.002
Average HD, mm	3.6 [2.7–4.5]		
95% HD, mm	9.0 [6.7–13.0]		
Workflow duration			
Total duration, min	26 [24–29]	65 [61–70]	<0.001
Anatomy segmentation, min	12 [10–12]	38 [35–40]	<0.001
EAM and anatomy merge, min [IQR]	8 [7–9]	13 [12–15]	0.002
Target drawing, min	6 [5–7]	15 [11–16]	<0.001
Number of software crashes	0	7	0.05

All values are reported in median [IQR].

EAM, electroanatomical map; min, minutes; mL, millilitre; mm, millimetre; HD, Hausdorff distance.

### Interobserver agreement for CardTV-EP creation and transfer using single centre LUMC data

Datasets of 20 patients who underwent work-up for STAR in the LUMC were used (median age 65 years [IQR 57–75], 95% male, median LVEF, 32% [IQR 26–44%], 80% NICM, median number of ablations, 1 [IQR 1–3]). For reference, from January 2018 to March 2024, a total of 964 ventricular ablations were performed. All included study patients (*n* = 20) had undergone at least LV endocardial and aorta mapping. In 19 (95%) patients, the LM was tagged during the procedure for image registration. Eleven (55%) patients underwent additional RV and PA mapping. In all patients, an ECG-gated CT with LV contrast was present, and in 11/20 patients, LV and RV contrast was available. In addition, a planning CT was available in seven patients. See *Table 1* for details. The clinical AOI and additional three mock areas per patient were indicated on the 3D EAM, and CardTV-EP_inv_ were created and transferred independently by both observers on the ECG-gated CT scan. In one patient, only two additional mock areas were created because the mapping density in the basal anterior region was not sufficient to create an AOI.

A total of 79 CardTV-EPs were compared. There was no statistically significant difference between the overall volume sizes between observers (11.8 mL [IQR 10.1–13.7] for observer #1 vs. 10.7 mL [IQR 9.6–11.8] for observer #2 (*P*-value: 0.17)). Only for the mid-septal location, the volume size was statistically different between both observers (13 mL [IQR 9–16] vs. 10 mL [IQR 7–15], *P*-value 0.01). See *Table 3* for details.

**Table 3:** Interobserver comparison using ADAS for single centre data

CardTV-EP location	Observer 1	Observer 2	*P*-value
All CardTV-EP_inv_ (*n* = 79)			
Volume, mL	11.8 [10.1–13.7]	10.7 [9.6–11.8]	*0*.*17*
Difference in volume, mL	1.2 [1.1–1.5]		
Average HD, mm	1.7 [1.5–2.0]		
95% HD, mm	4.8 [3.5–5.7]		
Basal anterior (*n* = 19)			
Volume, mL	8.5 [6.8–14.6]	8.3 [7.4–12.2]	*0*.*36*
Average HD, mm	1.6 [1.2–2.1]		
95% HD, mm	4.2 [3.3–6.5]		
Apico-inferior (*n* = 20)			
Volume, mL	13.1 [10.0–18.2]	12.6 [8.3–17.5]	*0*.*10*
Average HD, mm	1.5 [1.0–1.8]		
95% HD, mm	4.2 [3.0–5.0]		
Mid-septal (*n* = 20)			
Volume, mL	12.6 [9.3–16.3]	9.6 [7.4–15.1]	*0*.*01*
Average HD, mm	1.8 [1.2–2.7]		
95% HD, mm	5.6 [3.6–7.3]		
Mid-lateral (*n* = 20)			
Volume, mL	13.8 [8.8–17.3]	13.0 [9.9–17.6]	*0*.*91*
Average HD, mm	1.8 [1.3–2.3]		
95% HD, mm	4.6 [2.8–6.5]		

All values are reported in median [IQR]. See *Table 1* or *Table 2* for abbreviations.

*P*-value denoted in italics.

CardTV, cardiac target volume.

The average HD between all volumes created by the two observers was only 1.7 mm [IQR 1.5–2.0], and the 95th percentile distance was 4.8 mm [IQR 3.5–5.7]. Agreement was highest for the apico-inferior volumes (average HD 1.5 mm [1.0–1.8]), and lowest for mid-septal volumes (average HD 1.8 mm [IQR 1.2–2.7]). See *Table 3* for details.

The presence of left-sided or left- and right-sided contrast did not influence the interobserver agreement: the average distance was 1.9 mm [IQR 1.4–2.1] in patients with only left-sided contrast and 1.7 mm [IQR 1.4–1.9] in patients with left- and right-sided contrast (*P*-value: 0.552).

From six patients, the CardTV-EP_inv_ were created and transferred using both the ECG-gated CT and the non-gated radiotherapy planning CT. The median difference between volumes was 4.0 mL [IQR 2.5–9.5], where the non-gated scan always showed the larger of the two volumes (median volume size ECG-gated scan, 34 [IQR 17–53] vs. 39 mL [IQR 29–59] in non-gated scan). The average HD between volumes was 2.9 mm [IQR 2.3–3.5].

### Interobserver agreement for CardTV-EP creation and transfer using multicentre data of Stereotactic arrhythmia radioablation-treated patients from the STOPSTORM.eu consortium

Data of 19 patients (median age 65 years [IQR 59–73], 90% male, 47% NICM, median LVEF 32% [IQR 26–44], median 2 prior VT ablations [IQR 2–4]) from four different centres (LUMC 7, CHUV 6, IKEM 3, MUMC 3) who were prospectively included in the STOPSTORM.eu registry and treated with STAR were processed by the two observers (*Table 1*). In all patients, the clinical CardTV-EP_inv_ was created, transferred, and treated. The quality of the EAM data (number of chambers/landmarks, completeness of surface mapping, point density) varied between cases. Left ventricular contrast was present in all but one patient (95%), and both LV and RV contrast was present in 10 patients (53%). The median difference in volume sizes between observers was 2.1 mL [IQR 1.1–5.2]. Hausdorff distance between volumes was a median 1.8 mm [IQR 1.5–2.7], and the 95th percentile distance was 5.4 mm [IQR 3.9–7.9]. In 11/19 patients, a minimum of three chambers/landmarks was mapped, and in 11/11 LV or LV/RV contrast was given. The median HD and 95th percentile distance in these patients were significantly smaller compared to patients with less than three chambers/landmarks mapped (median distance 1.6 mm [IQR 1.3–2.0] vs. 2.6 mm [IQR 1.8–4.1]; 95th percentile distance 4.1 mm [IQR 3.6–5.7] vs. 7.3 mm [IQR 5.3–11.3], both *P* < 0.05). See *Tables 4* and *5* for details.

**Table 4:** Interobserver comparison using ADAS for multicentre data

	Observer 1	Observer 2	*P*-value
CardTV-EP volumes (*n* = 19)			
Volume, mL	22.0 [12.9–38.0]	26.0 [13.0–43.4]	0.08
Difference in volume, mL	2.1 [1.1–5.2]		
Average HD, mm	1.8 [1.5–2.7]		
95% HD, mm	5.4 [3.9–7.9]		

See *Tables 1–3* for abbreviations. All values are reported in median [IQR].

**Table 5:** Interobserver variability in multicentre stereotactic arrhythmia radioablation-treated patients according to number of mapped structures

	All	≥3 structures mapped EAM	≤3 structures mapped EAM	*P*-value
	*n* = 19	*n* = 11	*n* = 8	
Average HD, mm	2.0 [1.6–3.1]	1.6 [1.3–2.0]	2.6 [1.8–4.1]	0.02
95% HD, mm	5.8 [4.1–8.5]	4.1 [3.6–5.7]	7.3 [5.3–11.3]	0.01

See *Tables 1–3* for abbreviations. All values are reported in median [IQR].

### Learning curve for the workflow

**Figure 1:**
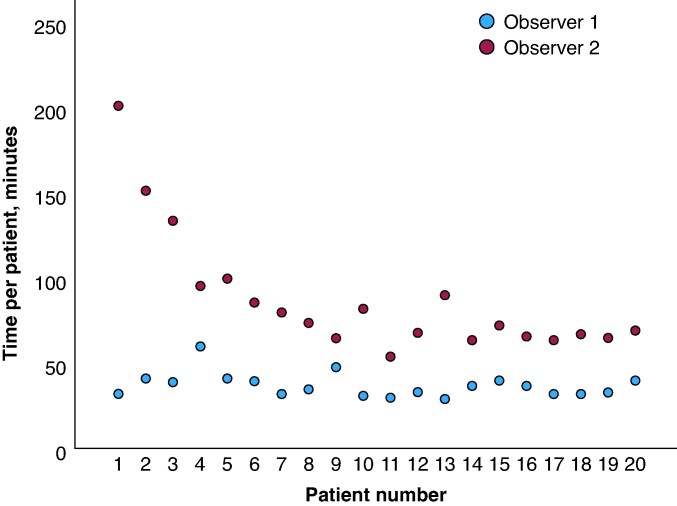
Workflow duration for consecutive datasets using ADAS for experienced and inexperienced observers.

Following the one-time demonstration case using the written workflow instruction, the duration of the workflow for the inexperienced observer decreased significantly for consecutive treated patients: for the first five cases, observer #2 needed a median of 134 min [IQR 98–177] to complete the workflow, which dropped down to a median of 68 min [IQR 65–80] for the next 15 (*P* < 0.001). Linear regression analysis showed a statistically significant downward trend in workflow completion time for observer #2 (*R*^2^ 0.534, *P* < 0.001). The time needed by observer #1 to complete the workflow remained stable throughout the study (mean 37 ± 7 min (*R*^2^: 0.06, *P* = 0.155)). See *Figure 1* and Supplementary material online, *Table S1* for details.

The agreement between observers also improved over time. For the first five cases, the HD between volumes was 2.2 mm [IQR 1.7–3.0] and 1.7 mm [IQR 1.3–1.9] for the following consecutive cases (*P* = 0.01). Similarly, the 95% percentile HD was 6.0 mm [IQR 4.6–8.5] for the first five cases and 5.0 mm [IQR 3.5–5.3] for the next 15 cases (*P* = 0.003). Linear regression analysis showed improvement over time for both average HD and 95th percentile HD between observers (average HD: *R*^2^: 0.355, *P*-value 0.01; 95th percentile distance: *R*^2^: 0.271, *P*-value 0.02). For the last 15 cases, 14/15 (93%) patients had an average HD between volumes of <2.0 mm. See Supplementary material online, *Table S1* and Supplementary material online, *Figures S2* and *S3* for details.

## Discussion

This study is the first to systematically analyse the accuracy, ease of use, and workflow duration for CardTV-EP_inv_ delineation for STAR using currently available software packages for single centre, mapping data acquired according to current recommendations^16^ (i.e. more than three structures mapped) and for multicentre datasets (with varying number of structures mapped) of STAR-treated patients from the STOPSTORM.eu registry.

The main findings of the study can be summarized as follows:

1. Both software packages, Slicer 3D and ADAS, allow for CardTV-EP_inv_ creation with complete overlap of the smaller volume, but resulting in larger volumes using Slicer 3D. Even if performed by an experienced operator, the Slicer 3D workflow is more time-consuming and vulnerable to software crashes.
2. For high quality EAM data acquired according to current recommendations^16^ (e.g. sufficient mapping density, number, and type of chambers/landmarks mapped), the interobserver agreement using the ADAS workflow is excellent with an HD between volumes of only 1.7 mm.
  The high interobserver agreement could be achieved with the ADAS workflow by an inexperienced observer after only five cases, with a shorter workflow duration than that of Slicer 3D by an experienced operator.
3. For unselected multicentre data from the prospective STOPSTORM.eu registry, the interobserver agreement was also high (average distance 2.0 mm) using the ADAS workflow, but significantly more accurate for cases with more than three structures mapped.

These findings suggest that ADAS workflow adequately addresses the four steps of potential interobserver variability, resulting in high interobserver agreement, provided that sufficient structures are mapped as advised by the recent consensus document.^16^

### Existing workflows for CardTV-EP_inv_ creation

Delineation of the CardTV-EP_inv_ that is based on invasive endocardial or endo- and epicardial EAM data indicated by the electrophysiologist on a surface and which needs to be extrapolated to a transmural volume and its transfer to the planning CT scan remains a manual task. Different workflows have been proposed using in-house-developed software and/or open-source software packages.^17,20,21,30^ Two-dimensional free-hand target delineation using eyeballing has proved to be inconsistent and poorly reproducible, resulting in large variation of CardTV-EPs.^31^ As invasive mapping data and cardiac CT data both provide 3D co-ordinates of anatomical structures and AOI, the advantages of workflows that utilize 3D to 3D registration are obvious. Of note, as of now, none of these software packages are CE-marked.^32^

Two single centre studies have analysed the Slicer 3D workflow (open-source software) for interobserver variability for steps 1–3 (see Methods section) of the data transfer to the radiotherapy planning CT.^17,21,27^ Both studies reported a high interobserver agreement after registration of the segmented anatomy of either the 3D location of the endocardial EAM tags without projection, or the 2D area created by connecting these non-projected tags. However, high agreement between endocardial tag locations or a fixed 2D area may not reflect agreement in 3D target volumes and location. Differences in wall thickness estimation and direction of transmurality in particular at the RV/LV insertion areas or at papillary muscle sites may influence the final volumes.

### Comparison between ADAS and Slicer 3D workflows

The ADAS 3D anatomy segmentation tool is a commercially available software package, which is used as a (pre-)procedural image integration tool for both atrial and ventricular ablations. To the best of our knowledge, there are no studies on the interobserver agreement for CardTV-EP_inv_ delineation using ADAS. The workflow for CardTV-EP_inv_ delineation using ADAS has been only case-reported.^26^

If performed by an experienced observer, the agreement on CardTV-EP_inv_ location created in ADAS and Slicer 3D was comparable to the previously reported interobserver agreement on target volume location using Slicer 3D.^21^ Of note, volumes created in Slicer 3D were larger than volumes created in ADAS, which explains the difference in HD despite a very good visual overlap of the volumes. The larger volumes can be explained by the workflow steps to create transmural volumes. In ADAS, the LV and RV endo- and epicardial contours are segmented separately, resulting in a 3D shell with fixed boundaries in which the CardTV-EP_inv_ can be created. In Slicer 3D, this function does not exist, which might lead to the extension of the volume towards the epicardium for free wall targets and the blood pool for septal targets and requiring target volume editing by the radiation-oncologist. Additionally, in ADAS, the option to first connect all pre-defined tags to encircle the AOI can be done in 3D, directly on the endocardial surface. In Slicer 3D, there are no such options. This may result in the creation of a larger CardTV-EP_inv_ than intended. See Supplementary material online, *Figure S4* for examples. The limited inaccuracy in volume determination using Slicer 3D is also reflected by the differences between observers in volumes delineation in a previous study, where one observer created significantly larger volumes than the other.^21^ In the current study, no such volume differences occurred with ADAS. Whether the larger CardTV-EP_inv_ volumes as created by Slicer 3D would impact safety or efficiency of STAR needs further investigation. When defining the definitive target for STAR editing of the CardTV-EP_inv_ volume to obvious non-target areas (e.g. blood pool or extracardiac) as described in the International Committee for Radiological Units report 83 is therefore warranted.^33^

Prior studies did not report on workflow duration, ease of use, or software crashes using Slicer 3D, which is important for clinical applications. The Slicer 3D workflow is open-source and available without costs. However, compatibility with software updates in the mapping systems and software stability are not guaranteed. Programming skills in Python are required to convert the data exported from the mapping system to the input data for Slicer 3D. The Slicer 3D software, with all necessary plugins installed, was prone to software crashes and freezes in our study, demonstrated by the high number of crashes leading to data loss in seven instances spread over four cases, whereas no software crash was observed with ADAS.

From a radiotherapy planning perspective, ADAS can output RTSTRUCT files, which is considered an industry DICOM standard. As of now, the Slicer 3D workflow only outputs CT scans with whitened out voxels containing the target area. For the radiotherapy planning, this is less desirable since manual overriding of the DICOM data is necessary.

## Influence of the quality of 3D mapping data

Acquisition of mapping data is at the discretion of the operator and may vary across centres. Patients referred for STAR are refractory to conventional treatment by definition. The mapping data are often obtained during a failed ablation, and patients may be electrical unstable that may impact the quality of data. Therefore, we have evaluated the interobserver agreement for CardTV-EP_inv_ delineation in patients treated at a single centre where a minimum of three chambers/landmarks were mapped in almost all patients, and in patients included in the prospective STOPSTORM.eu registry. According to the recent clinical consensus statement, it is advised to obtain detailed EAM covering the surface of the chamber of interest with anatomical marking of at least three chambers/landmarks in preparation of CardTV-EP_inv_ whenever possible.^16^ This advice is based on one prior study using the Slicer 3D workflow.^21^ In the single centre data cohort of this study, all patients met these requirements and the interobserver agreement for CardTV-EP_inv_ in this cohort was high. In the multicentre data, 24/32 patients met the advised mapping requirements. The high interobserver agreement using the ADAS workflow was confirmed for multicentre data with excellent results if at least three structures have been mapped, further supporting the advice of the clinical consensus statement.

### Influence of substrate location on observer agreement

To analyse the effect of substrate locations on the interobserver variability in the creation of the STAR volumes, mock targets were created to simulate prevalent substrate locations in patients referred for STAR. In a previous study, the lateral wall substrates had the highest interobserver variability, causing authors to suggest that margins might need to be increased when targeting substrates in this location.^21^ In our study, all target locations performed similarly, with only the mid-septal region having statistically significant differences in volume size. The increased HD in the mid-septal volumes can be explained by the difference in size between the volumes. One possible explanation for the size difference may be the absence of right-sided contrast, which hampers the accurate delineation of the RV septal border. Since extension of the volume towards the RV side of the septum affects mainly the blood pool, this may have no consequences for the radiation planning and on organs at risk. However, the location of the moderator band needs to be considered. For the lateral and apico-inferior substrate locations, however, this might have consequences for the radiation planning, because of the proximity of organs-at-risk such as the stomach, bowels, and lungs.

### Planning CT scan vs. ECG-gated CT scan

Planning volumes for STAR are created on the radiotherapy planning CT scans. To determine the differences in volumes between the two scans, the volumes were created on both the ECG-gated scan and the planning CT scan. The volumes created on ECG-gated CT scans were smaller than those created on planning CT scans. However, differences were small with a median of 4 mL between volumes, for a median volume of 39 mL. In all but one patient, the smaller volumes based on the ECG-gated CT were completely included in the larger planning CT-scan volumes. The difference in volume may be explained by the lack of ECG-triggering. The contours of the endo- and epicardium are less sharply demarcated due to the acquisition during systole and diastole, which in turn suggests a larger myocardial volume than when the CT slices are only created during diastole. Additionally, the slice thickness is four times higher and pixel spacing doubled in the planning CTs compared to the ECG-gated CT scan.

### Learning curve for the ADAS workflow

By including one highly experienced observer and one inexperienced observer, this study can determine the learning curve of new users. The second observer had no experience in creating CardTV-EP_inv_ for STAR and in the use of ADAS anatomy segmentation. Thus, the outcomes for workflow duration and observer agreement improvement reflect a real-world learning scenario showing that a new user can accurately and reliably create CardTV-EP_inv_ after a low number of cases. Our study showed clear improvement of workflow duration and interobserver agreement. After five cases, almost all cases had an average distance shift between volumes below 2 mm. This robustness of the workflow may lead to improved patient outcomes because of the high accuracy of data transfer from the mapping system to the planning CT. The time needed for a case dropped significantly after five cases, from a median of 134 min for the first five to a median of 68 min for the last 15. The observer agreement and workflow duration improvement after only five cases is highly relevant since the use of STAR for refractory VT is still limited, even in high-volume VT ablation centres. Online tutorials and expert case reviews (through e.g. STOSTORM.eu) may facilitate implementation of the workflow in clinical practice.

### Limitations

This study sought to overcome limitations of prior studies by increasing the case numbers and adding one more substrate location to the analysis. The median volume of the mock CardTV-EP_inv_ was smaller than those reported in previous clinical cases. However, we feel that this does not impact the reproducibility of the high interobserver variability, since the smaller the volumes, the less likely it is that random variance causes volume overlap. Also, the data used for this study are obtained from high-volume VT centres, which may limit the generalizability of the results.

### Conclusion

Cardiac target volume creation was faster and more robust using ADAS compared to the custom Slicer 3D software, with an excellent interobserver agreement in both single- and multicentre data, provided that recommended mapping standards are applied. There was a steep learning curve for inexperienced operators using ADAS, which may be relevant, considering the limited use of STAR, even in high-volume VT ablation centres.

## Supplementary Material

euaf122_Supplementary_Data

## Data Availability

The data underlying this article will be shared upon reasonable request to the corresponding author.
