# Supplementary material for: Excellent interobserver agreement and steep learning curve for target volume delineation for stereotactic arrhythmia radioablation using a commercial software
Source: Europace. 2025 Jun 16;27(7):euaf122. doi: 10.1093/europace/euaf122 (PMC12247503; doi:10.1093/europace/euaf122)
Supplement: euaf122_Supplementary_Data [file euaf122_supplementary_data.zip › Supplemental workflow final.pdf]

GUIDE FOR CARDTV DATA TRANSFER TO CT USING ADAS

LEIDEN UNIVERSITY MEDICAL CENTER

Table of Contents

1 Creating 3D anatomical model 2
1.1 Automatic segmentation: LA, LV and AO 3
1.2 Threshold segmentation: RA, RV and PA 4
1.3 Automatic coronary artery segmentation 7

2 LV wall thickness 9

3 3D model and Carto anatomical map alignment 11
3.1 Refinement of the alignment 12

4 CardTV creation on CT 13
4.1 CardTV creation 14

About This File

This file was created for the benefit of all people using ADAS 3D for clinical or research purposes.
The entirety of the contents within this file are free for public use.

SECTION 1

Creating 3D anatomical model

First, the CT-data need to be imported to adas in a DICOM format. To do that, select: New Study, DICOM (Select the DICOM file that you want to import), CTA.

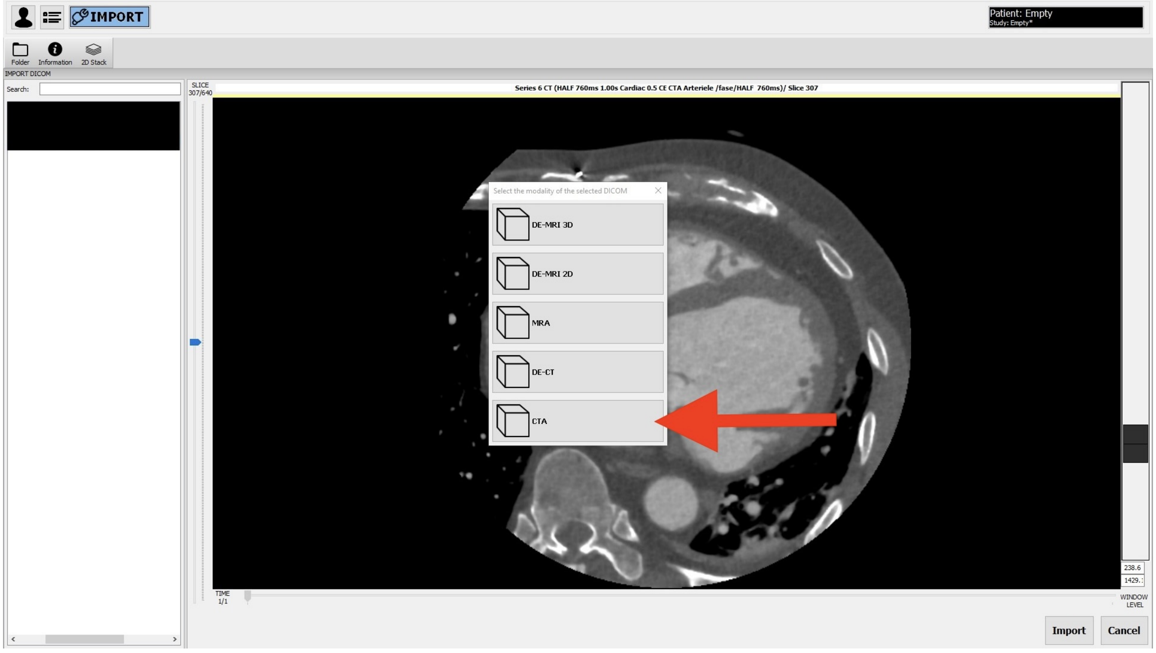

Figure 1: Select CTA when importing CT data.

Now, the mode 'Heart Anatomy Extraction' can be used to make the 3d anatomical model of the heart. The model will be created using 3 methods:

1. Automatic segmentation to extract the LA, LV and AO.
2. Threshold segmentation to extract the RA, RV, and PA.
3. Coronary arteries to add the LCA and RCA.

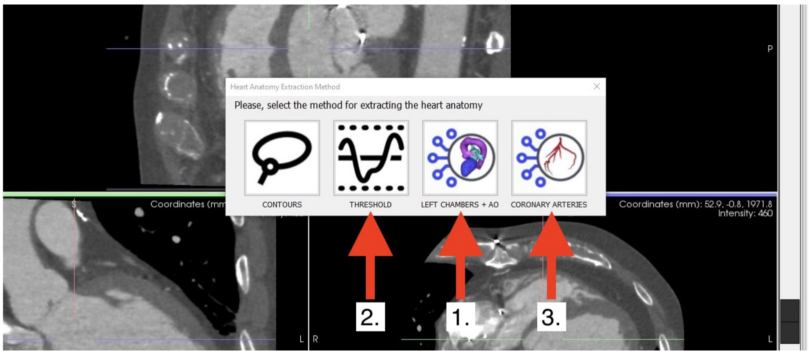

Figure 2: The different arrows indicate the modes that are used for this segmentation.

1.1 Automatic segmentation: LA, LV and AO

First select 'automatic segmentation' to extract the left atrium, left ventricle, and aorta (Figure 3).

- In some cases, depending on the quality and contrast distribution of the CT scan, the automatic segmentation may give inadequate results. In such cases, threshold segmentation can be used for the entire anatomy extraction.

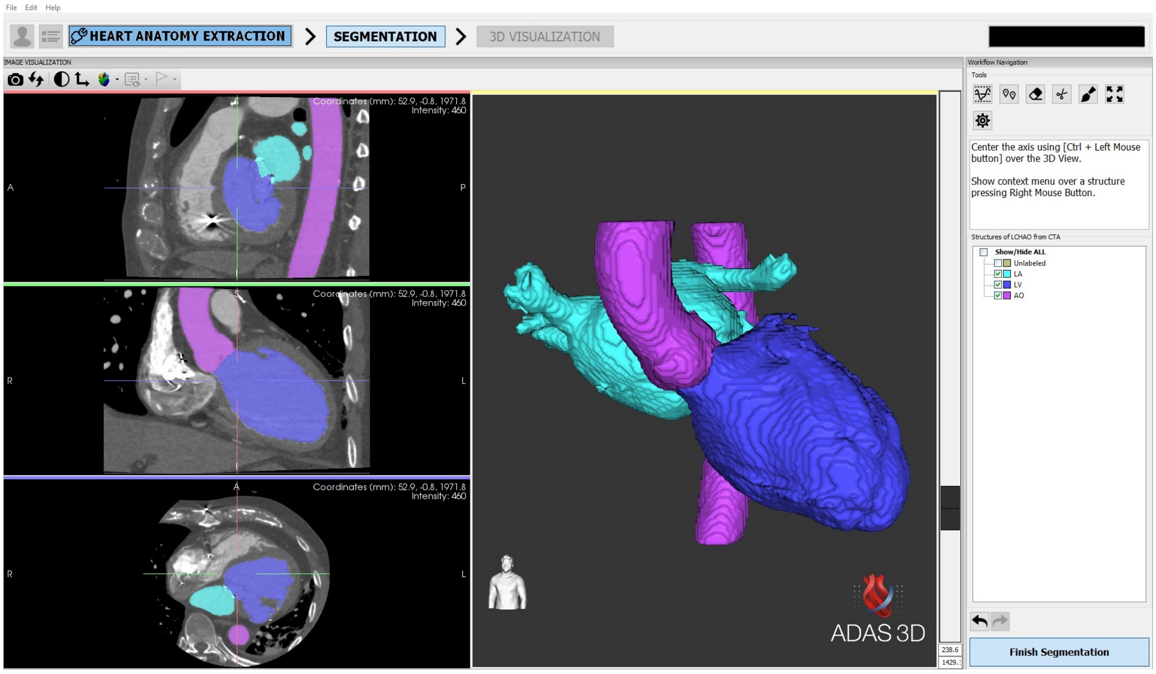

Figure 3: Constructed model from automatic segmentation

1.2 Threshold segmentation: RA, RV and PA

For this step, the thresholds must first be determined. The lower threshold should be placed at a point at which the ventricular septum is not included to the area of the right ventricle (Figure 4).

- If too much of the right ventricle is excluded this way, parts of the ventricular septum can be included and removed in the next steps.

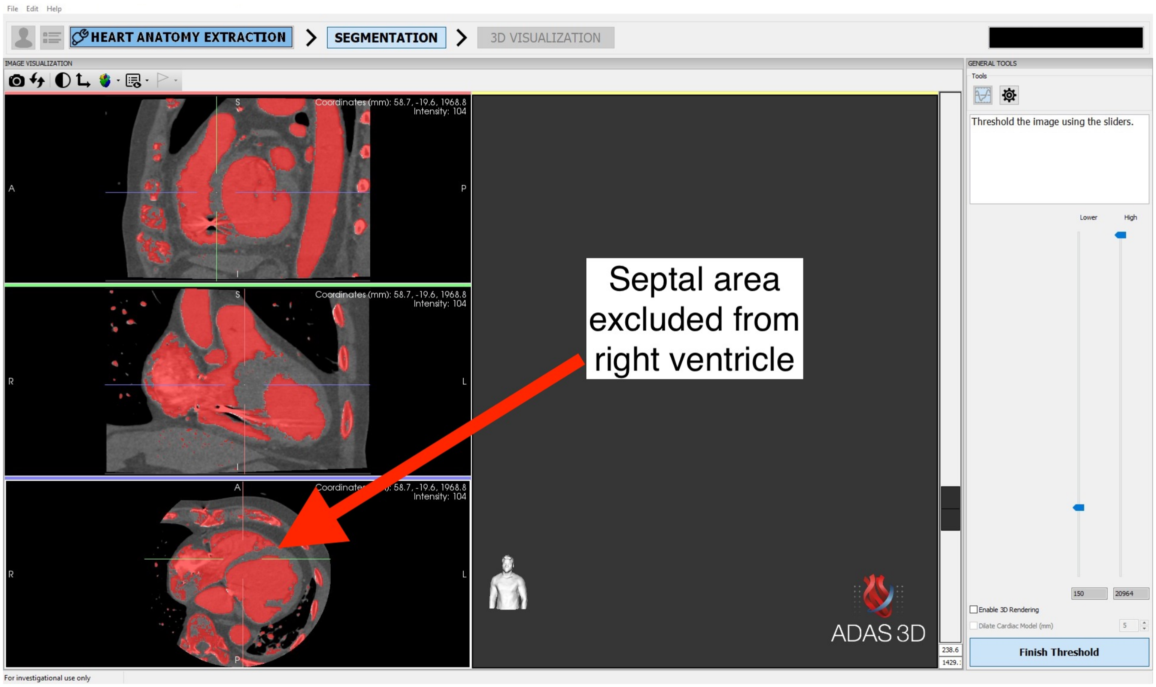

Figure 4: This image illustrates the right thresholds for manual segmentation.

The anatomical model should now resemble Figure 5. The eraser tool can be used to remove the various structures until adequate segmentation is reached (Figure 6).

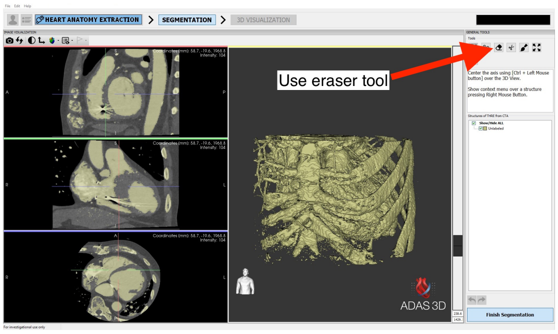

Figure 5: Model with all structures included

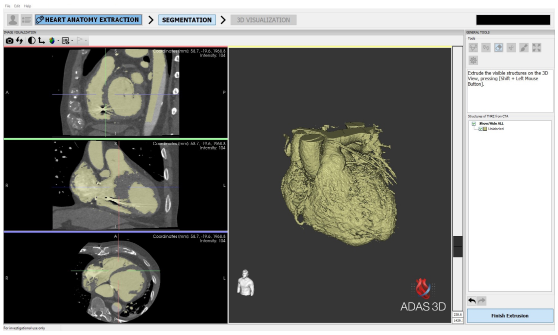

Figure 6: Large structures are removed using the eraser tool.

Next, use the seed function and place seeds on the LA, LV, AO asc, AO desc, RA, RV and PA. This will roughly divide the various anatomical regions as seen in Figure 7.

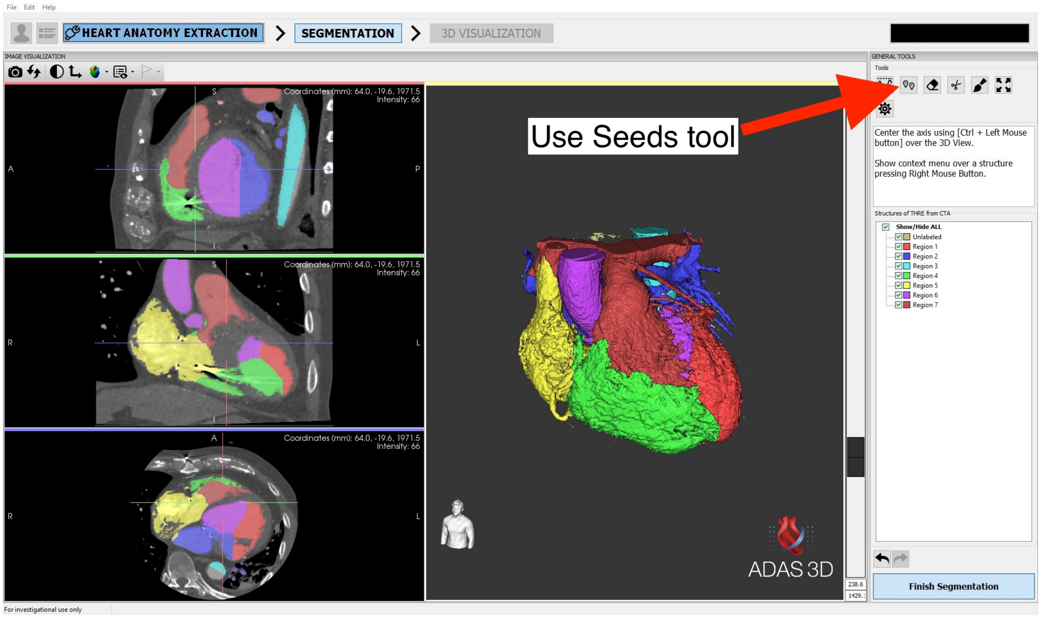

Figure 7: Segment heart in areas using the seeds tool (red arrow).

Remove the important regions (RV, RA, and PA) on the right window and determine on the CT view if any of the structures that need to be saved are part of the other structures (Figure 8). If any structures are still fused, use the split tool or paint tool to separate these regions.

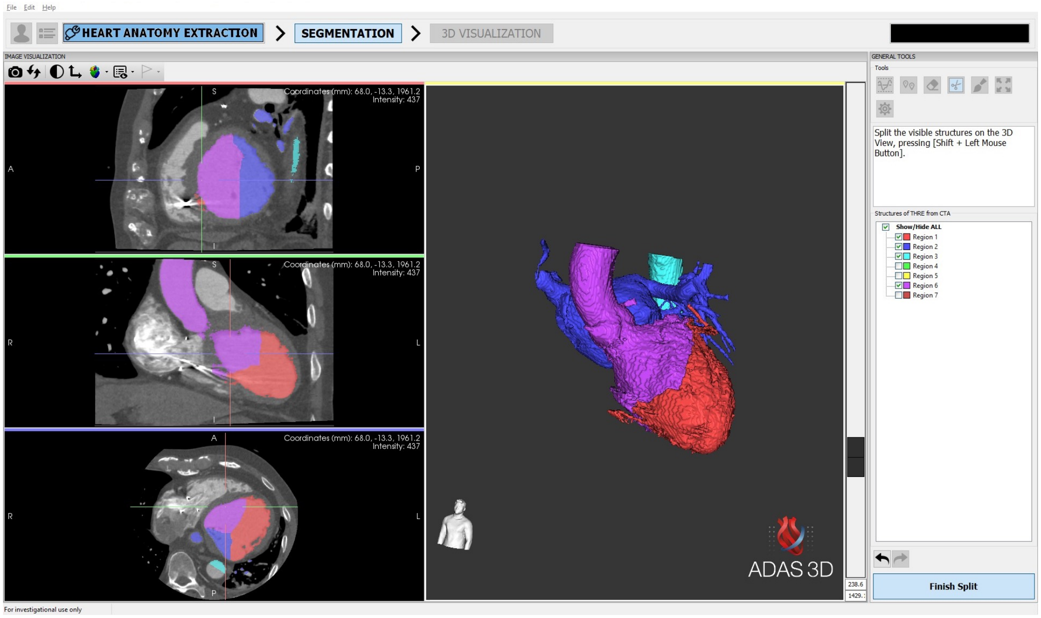

Figure 8: 3D visualization of segmented left-sided anatomy

Now, visualize relevant regions for this segmentation (RA, RV, and PA) to see if any non-cardiac or other cardiac structures are still present. In this case (Figure 9), part of the left ventricle is included in the right ventricle. Use the paint tool to select this area and remove it.

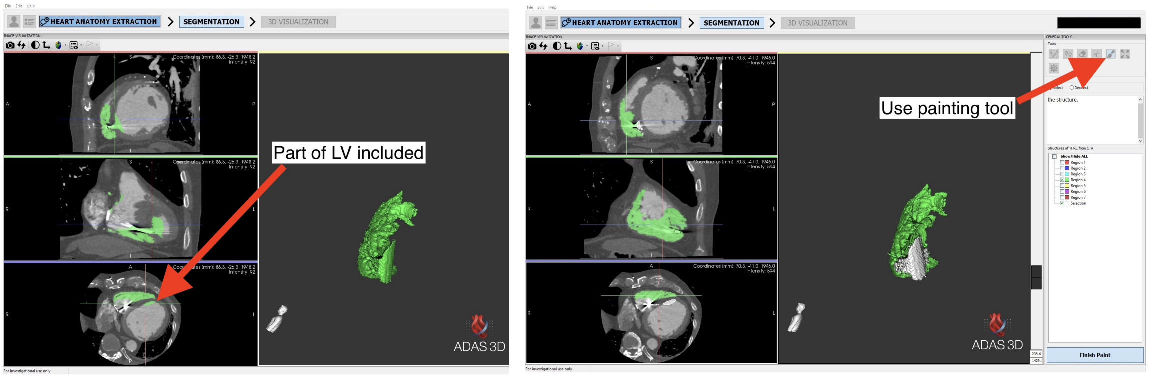

Figure 9: Image on right shows that part of the LV is included in the RV. Use the paint tool to select and remove this area.

Now, merge the right ventricle and the pulmonary artery. If necessary, parts of the right atrium can be separated from the right ventricle using the split or paint tool.

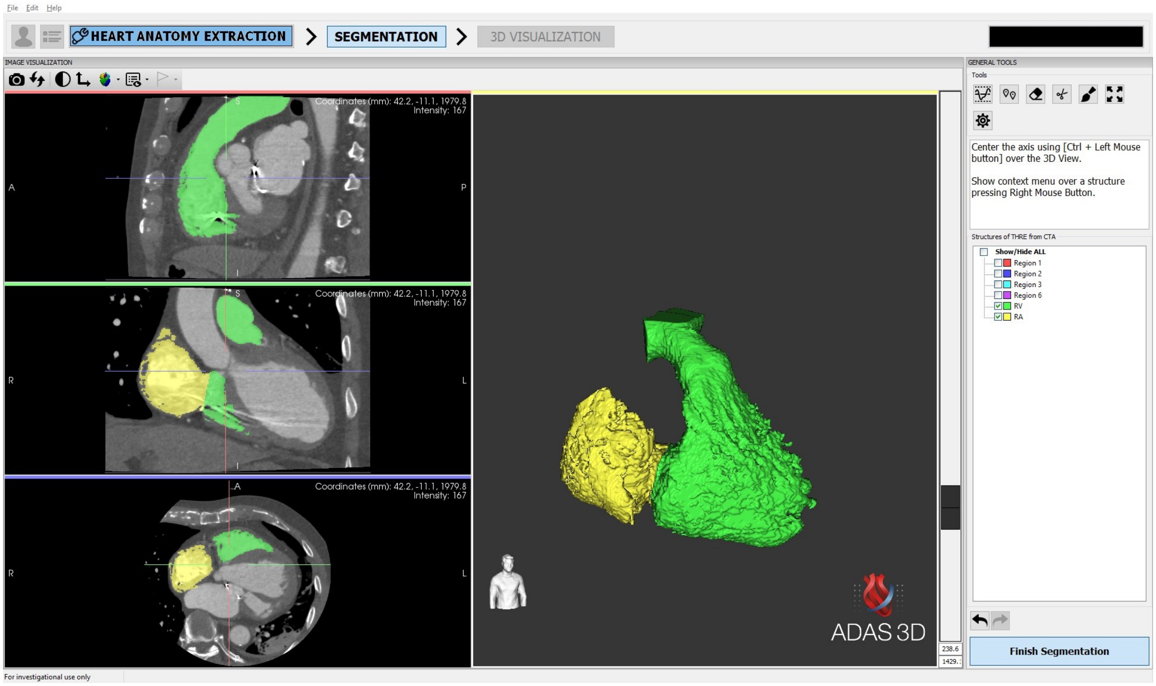

Figure 10: Final segmentation of the RA, RV and PA.

1.3 Automatic coronary artery segmentation

In this mode, all the coronary arteries will be automatically segmented.

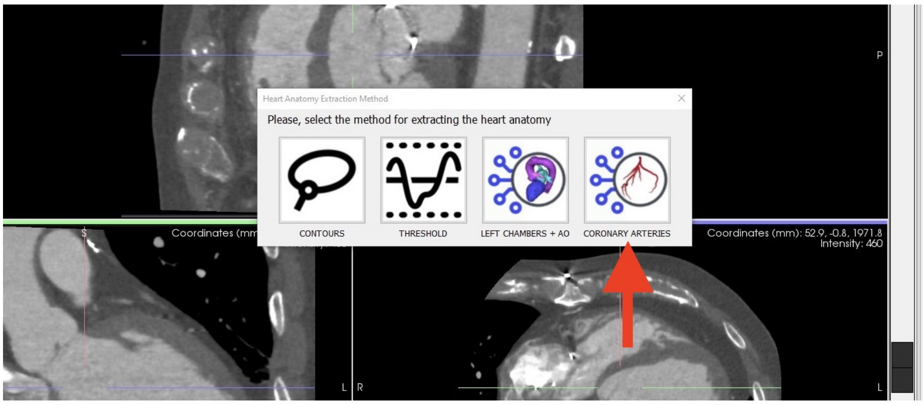

Figure 11: From Heart Anatomy Extraction, select the option on the right for coronary artery segmentation.

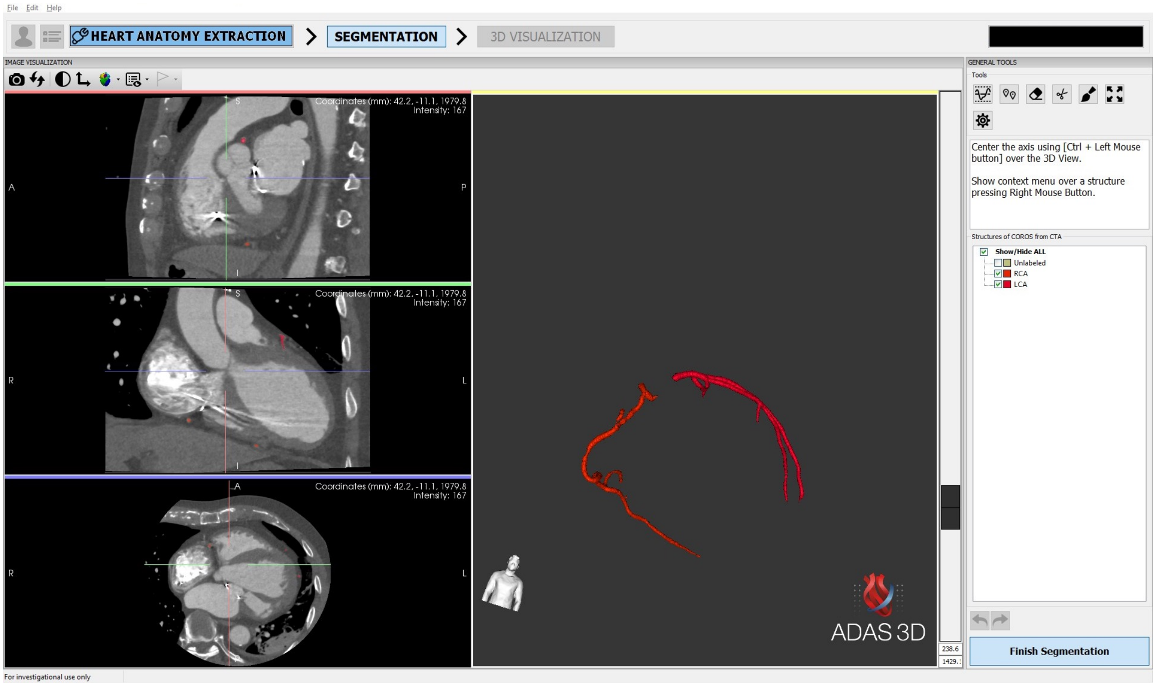

Figure 12: The coronary arteries after automatic segmentation

Now, open 3d visualization to see the constructed model of the heart.

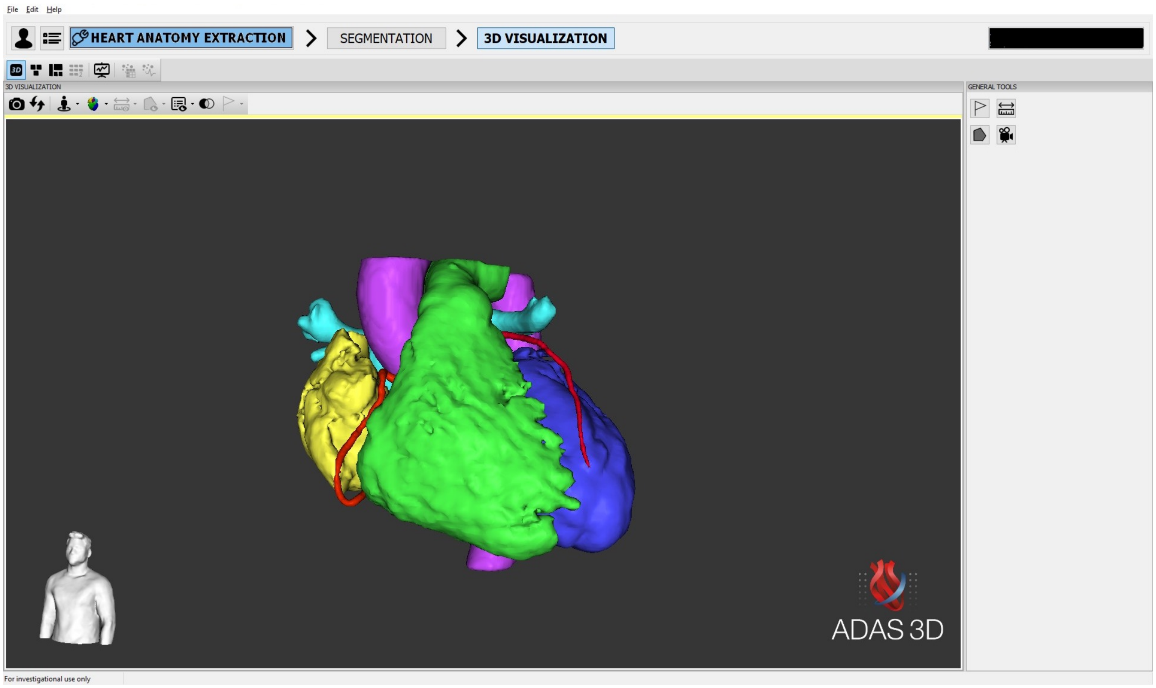

Figure 13: Final model of the 3d reconstruction of the heart.

SECTION 2

LV wall thickness

In this section, the wall thickness shell, bordered by the endocardial and epicardial contour is created. . This will eventually form boundaries of the target areas.

- Open the mode 'LV wall thickness' from the main menu.
- Start off by selecting 'Sparse Manual Contouring' (Figure 14). This mode enables the exclusion of papillary muscles from the endocardial surface.
- Place the anatomical landmarks.
- Draw the slice based adjustments as shown in Figure 15.

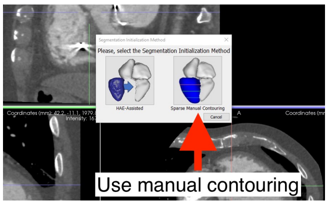

Figure 14: Choose the option on the right.

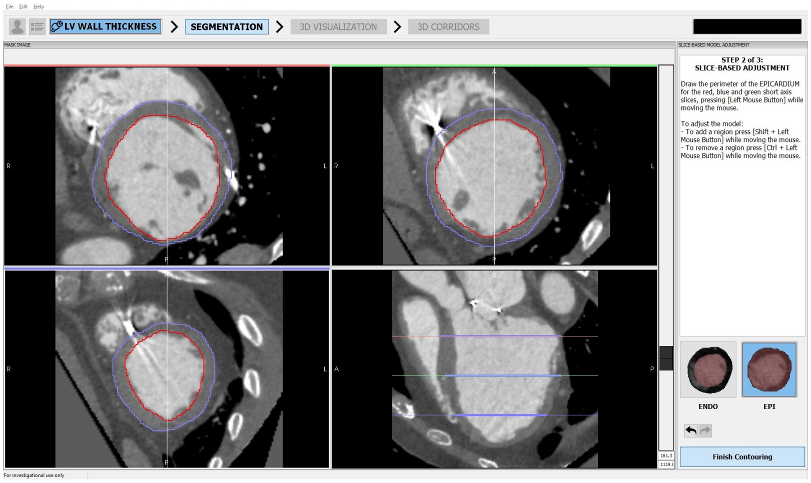

Figure 15

Next, contouring can be started.

- Start by moving the planes to visualize the short axis, horizontal long axis and vertical long axis views.
- Adjust the contours of the horizontal and vertical long-axis views.
- Make final adjustments in the short axis view.

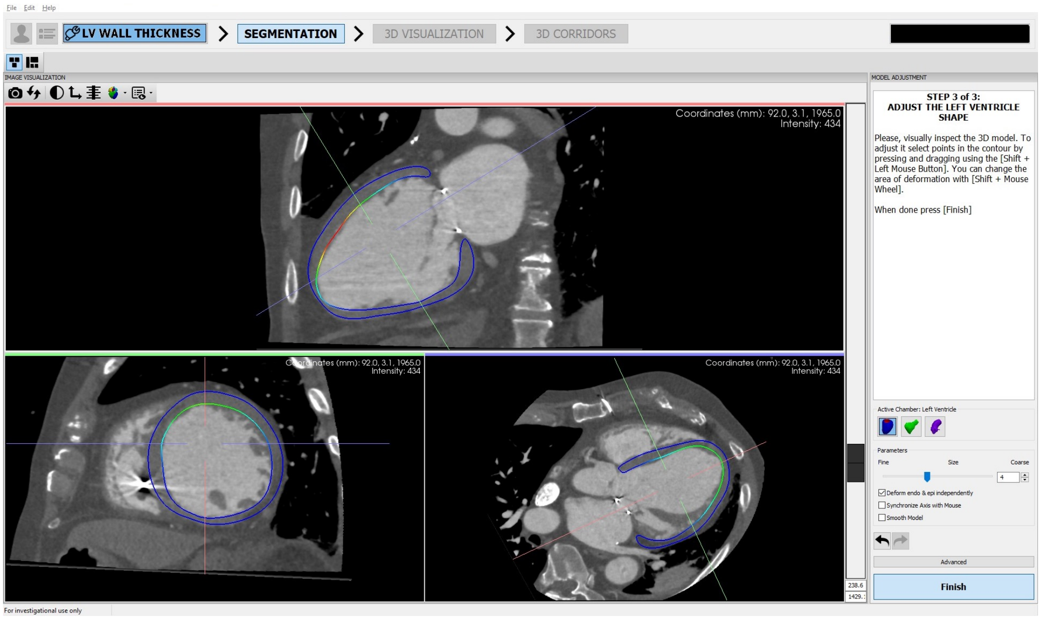

Figure 16: First, adjust the horizontal- and vertical long axis view (right and upper windows), then make small adjustments on the short-axis view.

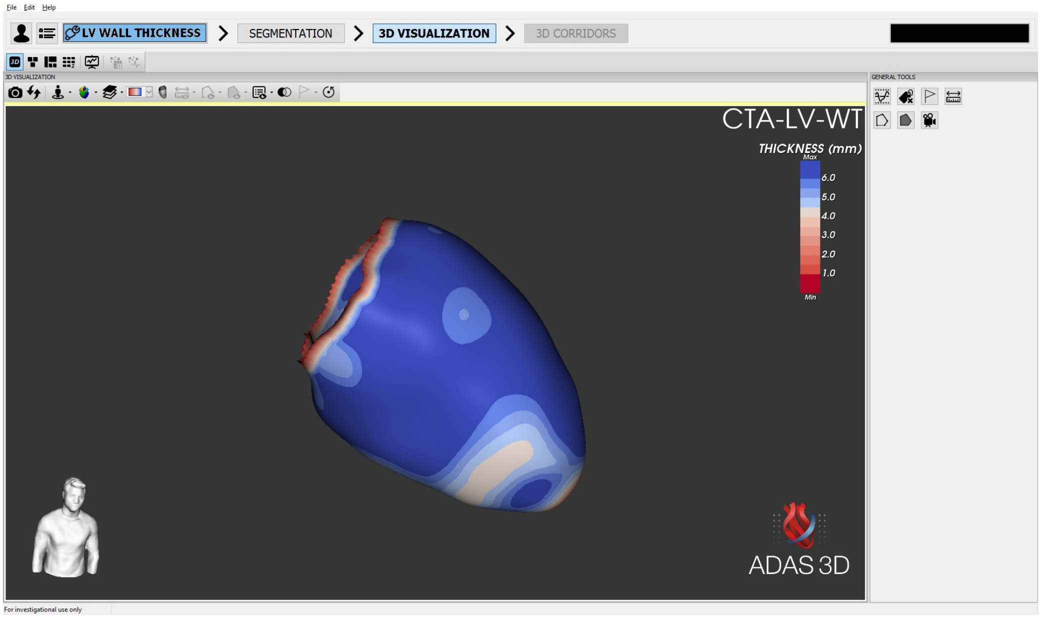

Figure 17: Final model

3

SECTION

3D model and Carto anatomical map alignment

Select the following options to start the alignment process.

- Import data
- EP study
- Select EP data
- Back to the start
- General tools
- EP study alignment

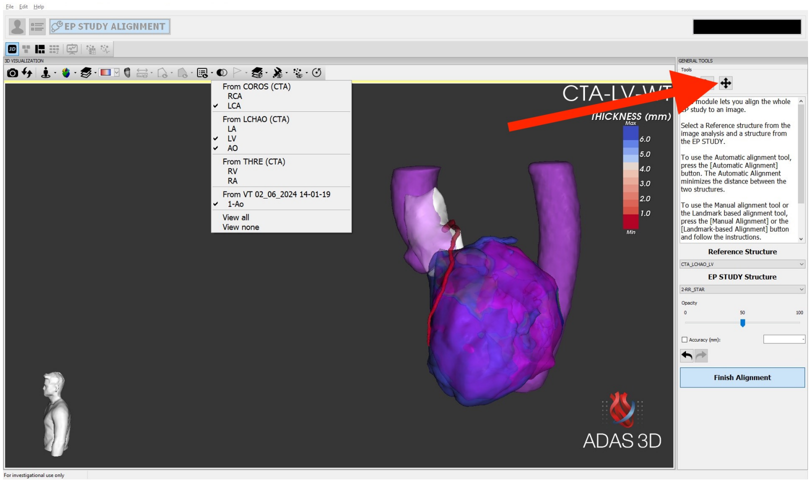

Figure 18: The model after aligning the structures approximately and visualizing various CTA structures.

Now, go to manual alignment (arrow Figure 18) and align the EP map and CT scan by approximation.

3.1 Refinement of the alignment

Take and repeat the following steps for precise alignment.

1. From the CTA models, visualize the aorta, left ventricle and left coronary artery.
2. From the EP structures, visualize the aorta.
3. Align the aortic root of the EP map with the aortic root from the CTA structure.
4. From the EP structures, visualize the left ventricle.
5. Align the ventricles while keeping the aortic root aligned.
6. Repeat the process for other available structures (RV, RA, PA, LA).

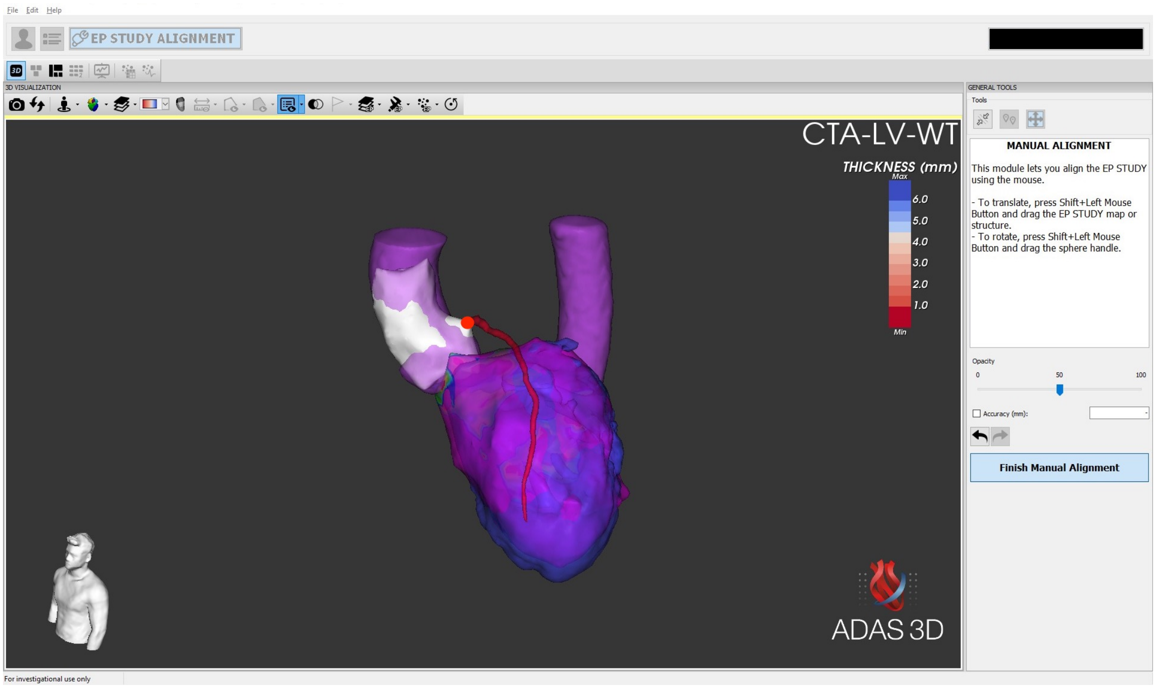

Figure 19: Correctly aligned EP map to the constructed 3D anatomical map. Note the three mapped structures in the window (left ventricle, aorta and left coronary ostium).

4

SECTION

CardTV creation on CT

From the main menu, open LV wall thickness.

1. Deselect all structures except the endocardium of the left ventricle.
2. Visualize the epicardium of the left ventricle (arrow 1 Figure 20).
3. Select 'Show EAM Points' and 'Project EP Points to layer' (arrows 2 and 3 Figure 20).
4. Go to 'EAM point tags' and visualize the desired region. In this case BA, but could also be STAR/CardTV etc. (arrow 4 Figure 20).

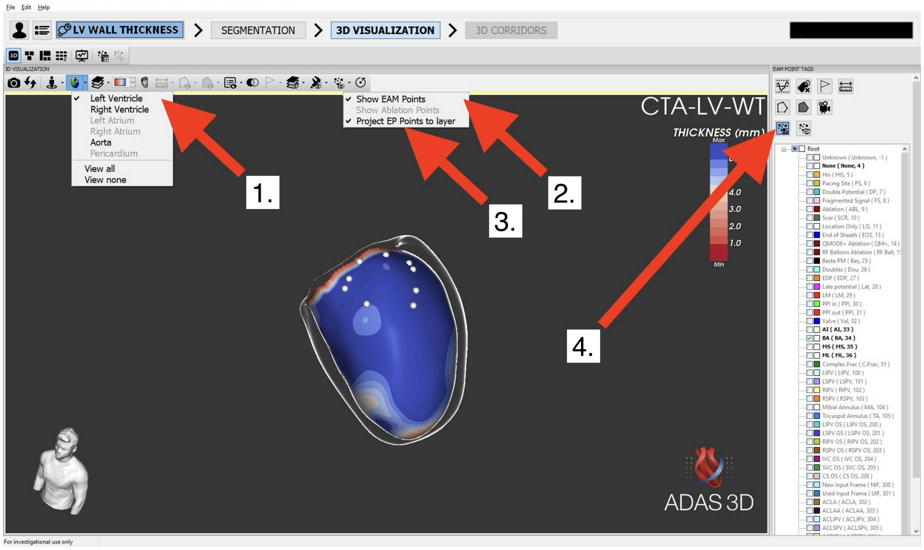

Figure 20: Arrow 1 highlights the mode to visualize the epicardial surface of the LV. Arrows 2 and 3 point to the window that makes the EAM points visible and project to the layers. Arrow 4 highlights to the mode to select the desired EAM points.

4.1 CardTV creation

1. Select the image region tool (arrow Figure 21) and create a new region.

2. Draw a line through all the points in the form of a ring as illustrated in Figure 21. Use the same brush thickness as the EAM points.

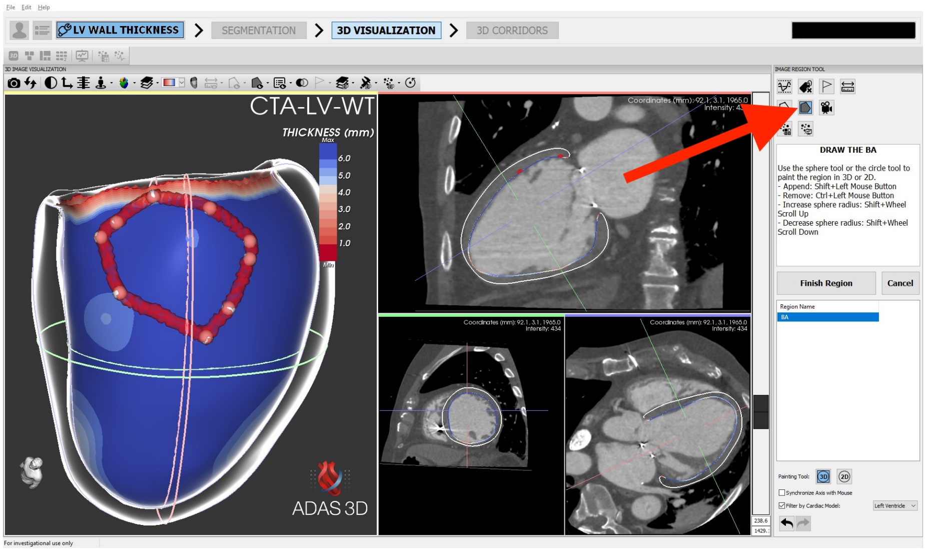

Figure 21

Now, create a new region, use a different color (left mouse click on the region) and create perpendicular lines from the endocardial ring to the epicardial surface (Figure 22).

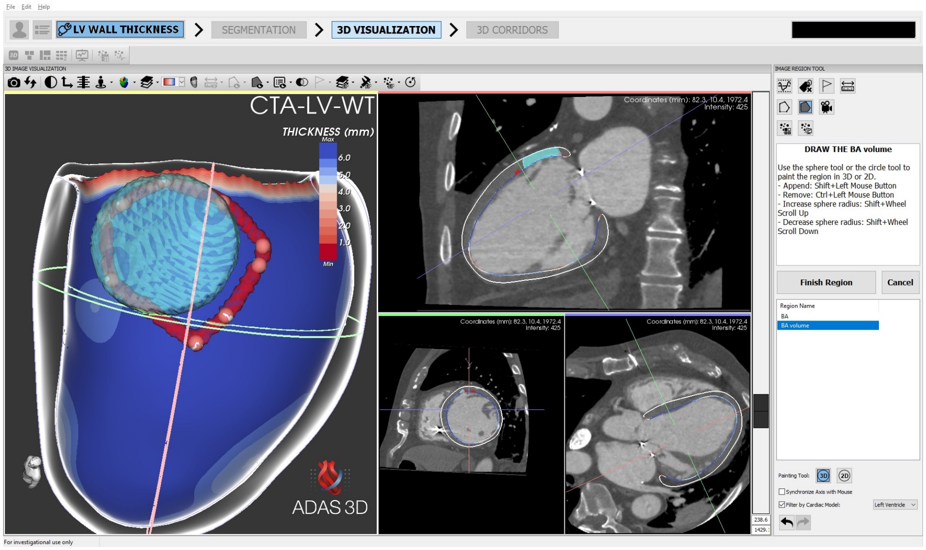

Figure 22: Area encircled by the red ring is partially filled. Use large brushes to fill the volume in and use smaller brushes to fill in the corners.

In some areas of the CardTV, the drawn region may not fill the complete heart wall.

- In such cases, use the region drawing tool on the CT scan itself (arrow Figure 23).

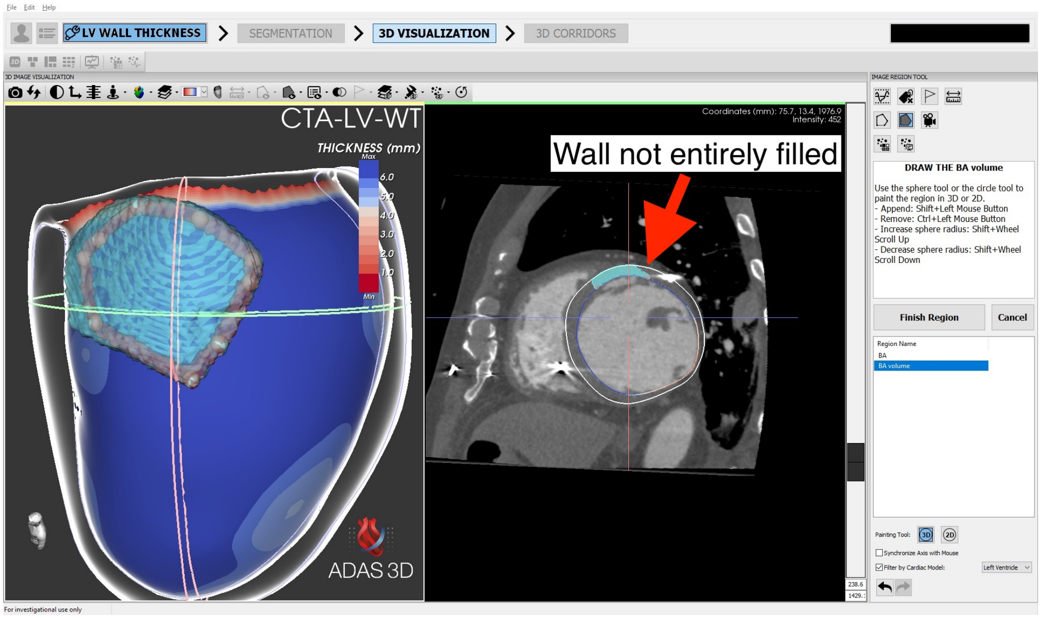

Figure 23: The CT plane on the right shows that the that the volume is not transmural from the endocardial circle. Use the brush on the CT plane directly to correct this.

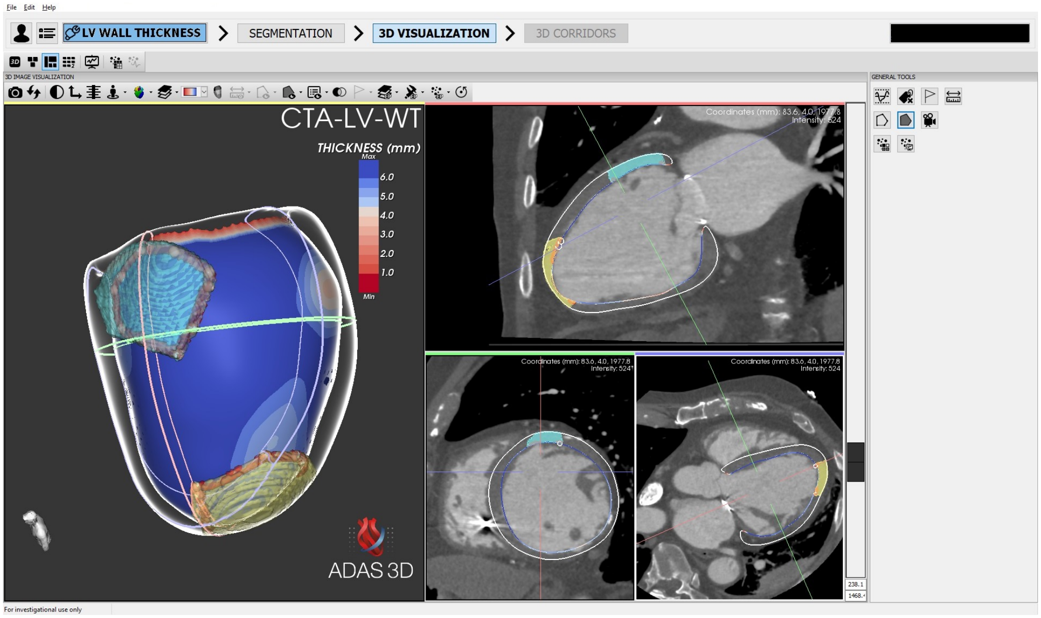

Figure 24: This image shows the final version of the CardTV. Different volumes can be made on the same model.
