# Supplementary material for: Excellent interobserver agreement and steep learning curve for target volume delineation for stereotactic arrhythmia radioablation using a commercial software
Source: Europace. 2025 Jun 16;27(7):euaf122. doi: 10.1093/europace/euaf122 (PMC12247503; doi:10.1093/europace/euaf122)
Supplement: euaf122_Supplementary_Data [file euaf122_supplementary_data.zip › Supplemental tables and figures_Second_Rebuttal.docx]

**Supplemental material**

**Supplemental Figure S1: Composition of patient cohorts**

STAR, stereotactic arrythmia radioablation; CardTV, Cardiac target volume

**Table S1: Workflow duration and interobserver agreement for first five and last fifteen cases for single center data**

| **Variable** | **First 5 cases** | **Last 15 cases** | **P-value** |
| --- | --- | --- | --- |
| **Workflow duration** |  | |  |
| *Workflow time duration observer #1, minutes* | 41 [36 – 51] | 33 [32 – 40] | 0.07 |
| *Workflow time duration observer #2, minutes* | 134 [98 – 177] | 68 [65 – 80] | <0.001 |
| **Interobserver agreement** |  | |  |
| *Average HD all volumes, mm* | 2.2 [1.7 – 3.0] | 1.7 [1.3 – 1.9] | 0.01 |
| *95% HD all volumes, mm* | 6.0 [4.6 – 8.5] | 4.5 [3.5 – 5.3] | 0.003 |
| *Average HD basal-anterior (BA), mm* | 2.2 [1.6 – 3.5] | 1.6 [1.2 – 2.0] | 0.03 |
| *95% HD basal-anterior (BA), mm* | 6.2 [4.1 – 8.4] | 4.0 [2.8 – 5.9] | 0.03 |
| *Average HD apico-inferior (AI), mm* | 1.8 [1.2 – 2.1] | 1.5 [1.0 – 1.7] | 0.58 |
| *95% HD apico-inferior (AI), mm* | 4.6 [3.2 – 9.1] | 3.7 [ 3.0 – 2.5] | 0.25 |
| *Average HD mid-septal (MS), mm* | 2.6 [1.8 – 3.4] | 1.6 [1.2 – 2.5] | 0.04 |
| *95% HD mid-septal (MS), mm* | 7.4 [5.0 – 8.6] | 4.8 [3.2 – 6.6] | 0.05 |
| *Average HD mid-lateral (ML), mm* | 2.5 [1.8 – 3.5] | 1.5 [1.2 – 2.2] | 0.01 |
| *95% HD mid-lateral (ML), mm* | 6.8 [4.2 – 9.4] | 4.5 [2.8 – 5.8] | 0.17 |

HD, Hausdorff distance. All values are reported in median [IQR]

**Figure S2: Mean HD for consecutive patients**

P=0.01, R^2^ 0.36

**Figure S3: Mean 95% HD for consecutive patients**

P=0.02 R^2^ 0.27

**Supplemental Figure S4: Typical examples showing the difference in volume size using Slicer and ADAS**

These examples show the expansion of the volume towards the blood pool and epicardium (left panel) and beyond the epicardium (right panel) using the Slicer workflow (orange volume) compared with the ADAS workflow (blue volume)
